# An Observation Data Driven Simulation and Analysis Framework for Early Stage C. elegans Embryogenesis

**DOI:** 10.4236/jbise.2018.118018

**Published:** 2018-08-28

**Authors:** Dali Wang, Zi Wang, Xiaopeng Zhao, Yichi Xu, Zhirong Bao

**Affiliations:** 1 Department of Electric Engineering and Computer Science, University of Tennessee, Knoxville, 37996, USA; 2 Environmental Science Division, Oak Ridge National Laboratory, Oak Ridge, TN 37831, USA; 3 Department of Mechanical, Aerospace and Biomedical Engineering, University of Tennessee, Knoxville, 37996, USA; 4 Developmental Biology Program, Memorial Sloan Kettering Cancer Center, NYC, USA

**Keywords:** C. elegans embryogenesis, Agent-based modeling, Deep reinforcement learning, Observation-driven modeling framework, 3D live images

## Abstract

Recent developments in cutting-edge live microscopy and image analysis provide a unique opportunity to systematically investigate individual cell’s dynamics as well as simulation-based hypothesis testing. After a summary of data generation and analysis in the observation and modeling efforts related to C. elegans embryogenesis, we develop a systematic approach to model the basic behaviors of individual cells. Next, we present our ideas to model cell fate, division, and movement using 3D time-lapse images within an agent-based modeling framework. Then, we summarize preliminary result and discuss efforts in cell fate, division, and movement modeling. Finally, we discuss the ongoing efforts and future directions for C. elegans embryo modeling, including an inferred developmental landscape for cell fate, a quasi-equilibrium model for cell division, and multi-agent, deep reinforcement learning for cell movement.

## 1. INTRODUCTION

**Figure 1.**
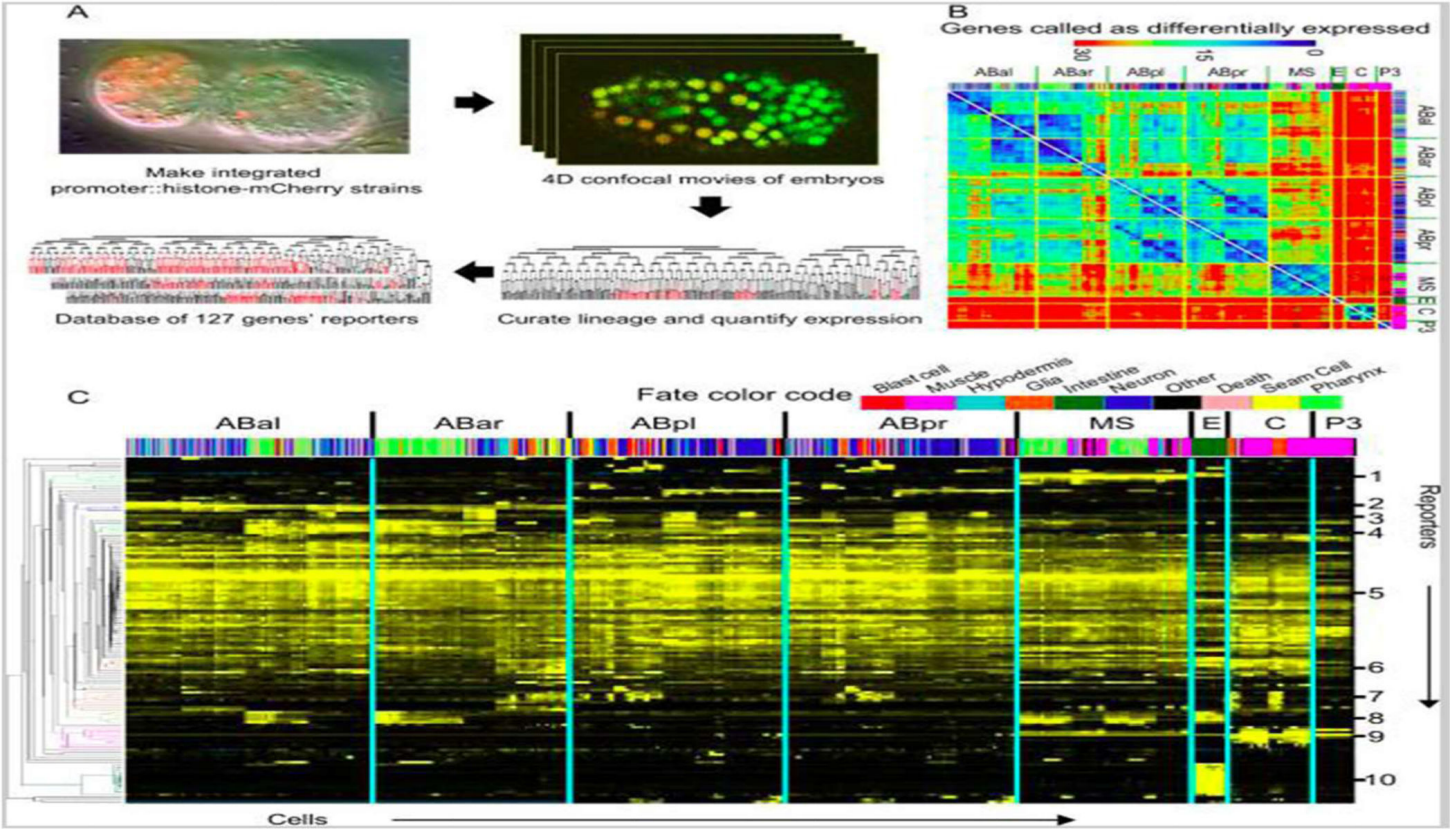
An illustration of data generation and usage in experiments. (A) Data collection strategy. (B) Heat map showing how many genes were expressed differently. (C) Expression patterns organized by hierarchical clustering. The original graph was published in [3].

Caenorhabditis elegans is a model organism widely studied in developmental biolo-gy. It is the first multicellular organism with known and invariant lineage [1]. In a room tem-perature, its embryos take only 13 hours to develop from 1 to 558 cells. C. elegans embryos are transparent and cylinder-shaped with roughly 50 μm long and a diameter of 30μm, which is easily accessible by microscopy. Genetic perturbation experiments can be as simple as feeding them with commercially available bacteria to target any gene. With all these advantages, C. elegans allows us to ask various bio-logical questions by generating large-scale microscopic data. We acquire two-channel 4D confocal images of live embryos on a Zeiss AxioObserver microscope frame with Zeiss 40x objective. The embryos are genetically modified with one fluorescence protein marking histone for tracking nuclei, and with an-other fluorescence protein providing additional biological information, such as protein localization and promoter activity. In our live-imaging system, we can perform three batches of experiments per day with 20~30 embryos per batch. During image acquisition, embryos are shot every 1 minute and in 30 slices to achieve high temporal and spatial resolution. In total, about 9,000 two-channel images can be accumulated daily, each of which contains 30 slices of double 512*512-pixel data. We have developed efficient algorithms to automat-ically trace every single cell over the course of early embryogenesis [2]. More importantly, powerful computational algorithms are needed to concentrate and extract information from the huge volumes of data, which is particularly challenged because many biological patterns are not intuitive to be formatted into a computer problem. A picture from a previous publication [3] is used here (Figure 1) to illustrate the data variety and complex in the laboratory experiments.

**Figure 2.**
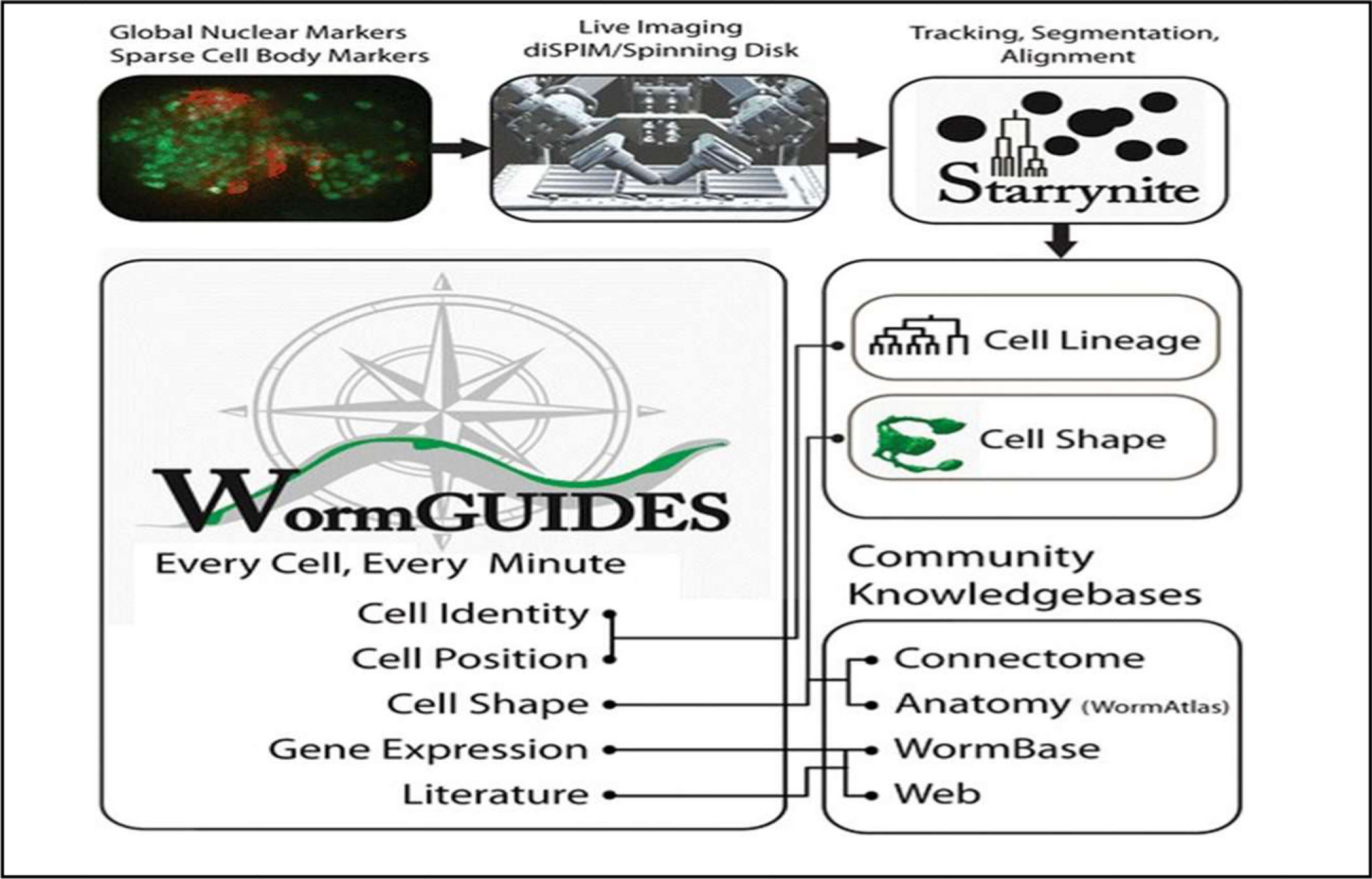
The major functions and data streams of WormGUIDES. The original picture was published in [4].

To facilitate the examination of cellular decisions in the developing nervous system of the nematode C. elegans, a consortium of biologists, computer scientists, and microscopists have worked together to create a novel systems-level resource for global understanding of C. elegans embryogenesis. A dynamic system, named Worm-GUIDES, was developed [4] to analyze the C. elegans lineage tree information on wild-type embryos or the embryos after gene mutation & manipulation. Worm-GUIDES also allow access to and visualize the connectome, the complete neural connectivity record which is uniquely available for C. elegans. More technical details on algorithm and applications related to WormGUIDES can be found in reference [5,6]. Figure 2 shows major functions and data streams used in WormGUIDES.

## 2. AN OBSERVATION-DRIVEN ANALYSIS AND SIMULATION SYSTEM

### 2.1. Agent-based Modeling Framework with Direct Data Assimilation

The massive 3D time-lapse live microscopy images allow biologists to systematically track individual cells in complex tissues and quantify cellular behavior over extended time windows. Therefore, it is not surprise that agent-based modeling (ABM) approach was adopted to study the embryogenesis. In an ABM framework, an individual cell can be modeled as an agent that contains a variety of information on its fate, size, division time, and group information. For an early stage C. elegans simulation, the cell fate, division, and movement can be directly derived from predefined observation datasets or represented by mathematical models (see the following sections). An example of this kind of agent-based model can be found in [7].

### 2.2. Cell Developmental Landscape for Cell Fate Modeling

C. elegans has a small number of somatic cells whose position and morphology are almost invariant from animal to animal. Because C. elegans is virtually transparent, cells can be identified in live animals using a simple bright-field microscopy technique, Nomarski differential interference contrast (DIC), or by expression of transgenic fluorescent reporter genes [8]. Since the 3D time-lapse imaging now used for imaging of metazoan embryo-genesis in different model organisms and tracking of individual cells, we can now directly track the whole cell lineage of C. elegans. The information can be directly used for the cell fate modeling of wildtype C. elegans. Furthermore, by combining automated lineage with tissue-maker expression-based assessment of cell types, we have recently shown that progenitor cell fates can be systematically assayed [9]. With the help of sequencing techniques, we can measure the mRNA content of individual cells that provide a more robust assay of cell types than using limited markers, with the apparent scalability to many cells. This research lead to a publication on how genes and gene networks shape the regulatory landscape and drive cells through the different trajectories of differentiation. It also provides a developmental landscape to model cell fate in complicated cases that involve gene mutation and manipulation.

### 2.3. Physical Model for Cell Division

There are many efforts that look into the mechanics of metazoan cell division using the C. elegans embryo as a powerful model system [10]. For example, some study used RNA to control the protein turnover that in turn influence the cell division. These mechanics happened as very fine scale and in a finite time period and our 3D time-lapse images are normally taken at much large time intervals (i.e. minutes). Therefore, we assume that mechanics plays an important role in regulating embryonic development. Many mathematical models have been developed to understand how the shape and growth of the embryo are influenced by various mechanical forces [11–14].

### 2.4. Machine Learning Model for Cell Movement

Cell movement in the early phase of C. elegans development is guided by gradients of various chemical signals, physical interactions at the cell-substrate interface and other mechanisms. If we treat the cellular movements as results of inherited and genetically controlled behavior regulated by inter- or intracellular signals, and these cell movements are also constricted by the neighbor cells and the eggshell, then we can use machine learning method to characterize the movement of individual cells within an embryonic system from 3D time-lapse images directly. This approach can be used to modeling the cell movement path in the early stage of C. elegans development where the regulation mechanisms are not well studied. We further assume that movement path of an individual cell is an optimal path that a cell can use to migrate under a collection of regulation networks and/or constraints within a physical environment. Then we transform the cell movement problem into a path optimization problem constrained by observation and predefined rules. An appli-cation of this approach to single cell direction movement is described in the following Section 3.3.

## 3. CURRENT RESULTS AND DISCUSSION

### 3.1. Cell Fate Representation for Wild-Type C. elegans

**Figure 3.**
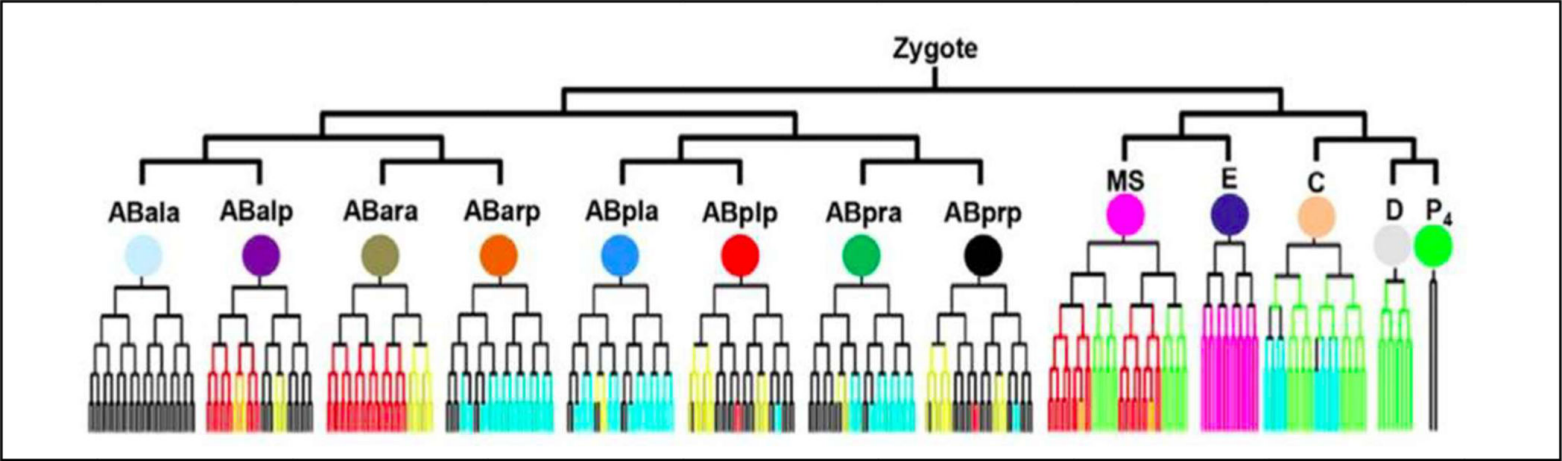
The illustration of lineage tree of wild type *C. elegans*. The color on branches represent when and where five tissue markers for pharynx (red), neuron (yellow), hypodermis (blue), muscle (cyan) and gut (magenta) are expressed in wild-type animals in a stereotypical manner. The division time is removed in the picture for a simple presentation. The graph was originally published in [9].

In our recently modeling efforts [7,15], the lineage of wild-type *C. elegans* (shown in Figure 3) are used to represent the fate of individual cells during the developmental process. Under these circumstances, the fate and also the division time are all predefined from the observation datasets from the 3D time-lapse live images.

### 3.2. A Simplified Physical Model for Cell Division

**Figure 4.**
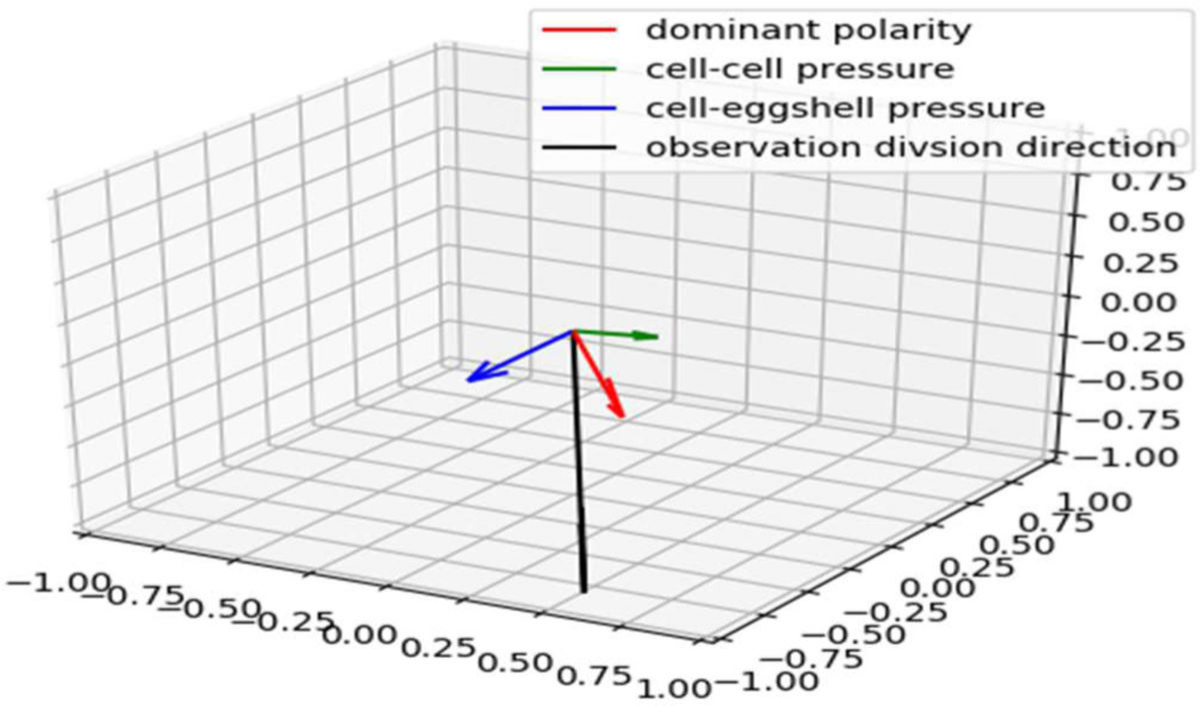
An illustration of the three components of the cell division direction and the actual observational direction.

A simple physical model was first developed to model the cell division direction. In an early attempt, we only consider three major components: 1) The direction of dominant cell polarity in the dividing cell; 2) The composition of cell-cell squeezes direction of force between the dividing cell and its neighbors [16]; and 3) The cell-eggshell squeeze direction force between the dividing cell and the eggshell (if there exists) [17]. As shown in Figure 4, we build a model for each part and get a number of samples. Each sample contains three 3D vectors that represent the three directions. We assign each vector a coefficient K as the parameter in the combined model. We transfer it into an optimization problem by minimizing the sum of the angle differences between the composition of the three simulation direction vectors and the actual observational division directions of cells.

### 3.3. Directional Single Cell Movement Simulation

**Figure 5.**
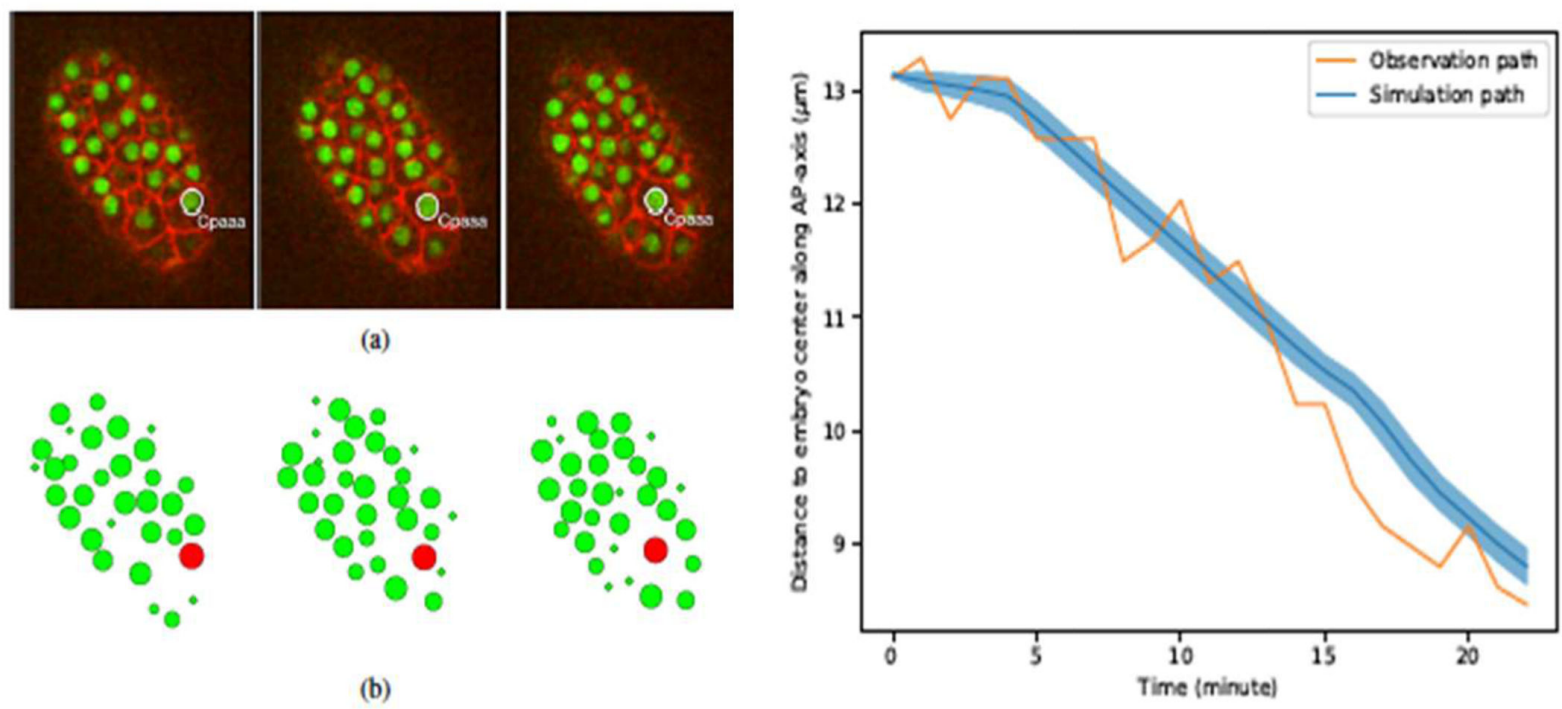
The observation and simulation result of Cpaaa cell movement (left) and the migration path of Cpaaa cell movement (right). The original pictures were submitted in [15].

In one recently work, we developed a method to model cellular movement using time-lapse images and deep neural networks to simulate the directional single cell movement within an agent-based modeling framework [15]. Directional cell locomotion is critical in many physiological processes during *C. elegans* development, including morphogenesis, structure restoration, and nervous system formation. We adopted deep neural networks to characterize the movement of individual cells within an embryonic system from 3D time-lapse images directly. We tested our model through two scenarios within real developmental processes, including a case of the anterior movement of the Cpaaa cell via intercalation, shown in Figure 5. The left graph shows the observation data (live image) and the simulation results of Cpaaa cell movement. The right graph shows the migration paths of Cpaaa. The simulation path is an average over 50 runs, and the shaded region indicates a range of one stand-ard deviation greater/less than the average value. We found that the movement path of Cpaaa is consistent with that in the 3D time-lapse images.

## 4. ONGOING EFFORTS AND FUTURE DIRECTIONS

### 4.1. Inferred Developmental Landscape for Cell Fate Representation

**Figure 6.**
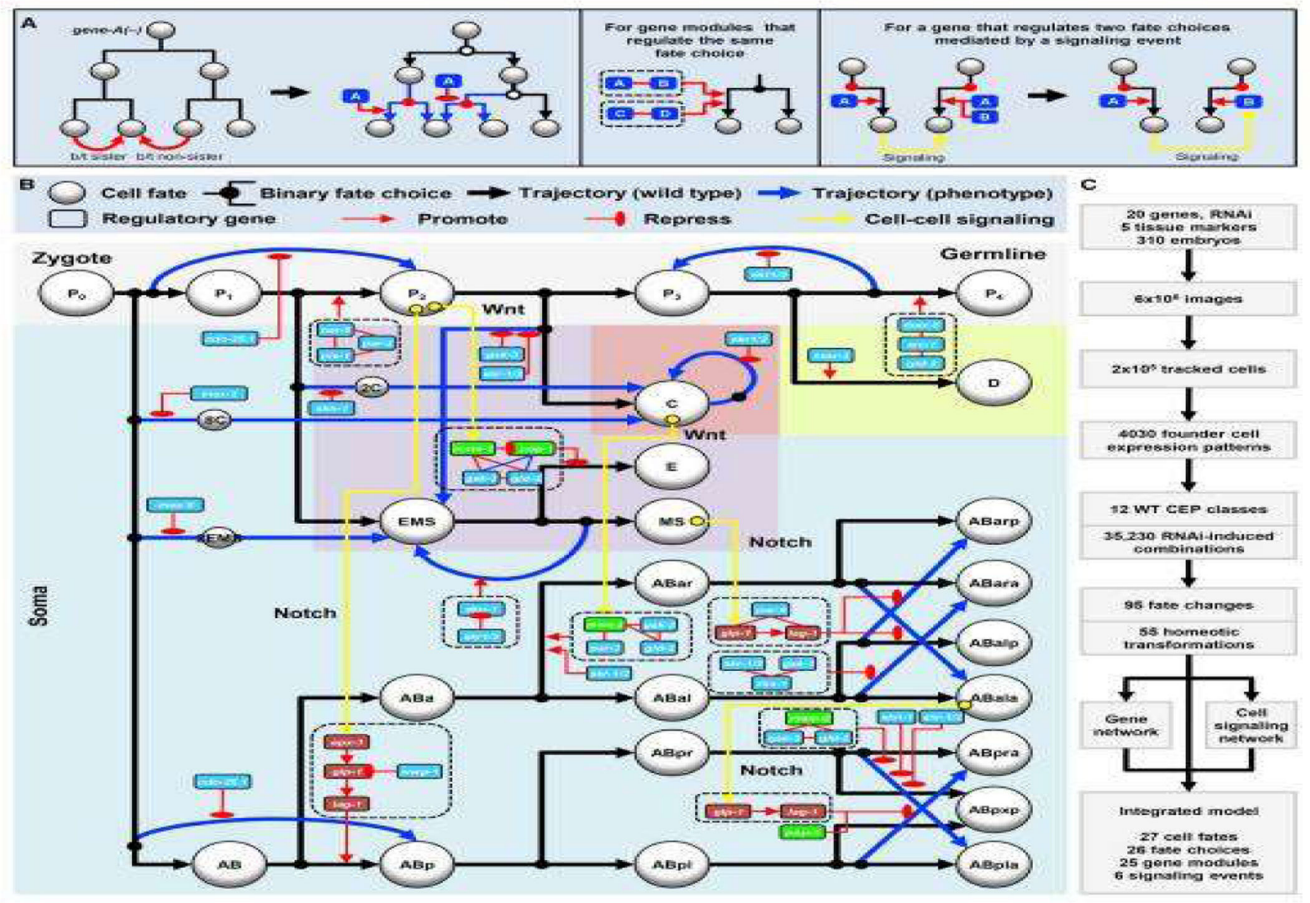
Inferred mechanistic model of development. This graph was originally published in [9].

In another previous work [9], a strategy to automatically infer mechanistic models of cell fate differentiation based on live-imaging data was developed using genetic perturbation ex-periment. We use cell lineage tracing and combinations of tissue-specific marker expression to assay progenitor cell fate and detect fate changes upon genetic perturbation. The analysis of the 3D time-lapse live images using cell lineage tracing and tissue-specific marker led to the construction a model for how fate differentiation progresses in progenitor cells and predict cell-specific gene modules and cell-to-cell signaling events that regulate the series of fate choices. By perturbing 20 genes in over 300 embryos, the experiments provided in-sights into gene function and regulated fate choice, including an unexpected self-renewal. As a result, an inferred mechanistic model of development was presented to elucidate how genes and gene networks shape the regulatory landscape and drive cells through the different trajectories of differentiation. Figure 6 shown a picture of an inferred developmental landscape for cell fate through gene mutation and manipulation of C. elegans embryos. This kind of developmental landscape then can be incorporated into our modeling framework to predict the cell fate under specific gene manipulation cases.

### 4.2. A Quasi-Equilibrium Model for Cell Division

Currently, we are developing a novel, simplified modeling approach to account for mechanical interactions among the cells during C. elegans embryonic development. Specifically, we represent each cell as a point mass and represent the interactions be-tween neighboring cells by spring forces. This simplified model is a versatile setup that can be conveniently integrated into the overall agent-based modeling framework. Moreover, the simplified modeling assumption allows us to explicitly track individual cells and easily account for the birth and migration of new daughter cells. Under this assumption, the embryo can be represented by a network of mass points connected to one another through springs. To first order approximation, we further assume that inertial forces and damping forces are negligible compared to the spring forces. Recall that, during the experiment, microscopic images are collected every minute to monitor the shapes and positions of the cells. Since the evolution of the network structure is a much slower process compared to the observation period, we assume the spring-mass network is in quasi equilibrium on the time scale of observation. To determine the positions of the cells, we calculate the potential energy of the mass-spring network. Since the network is in equilibrium, the cell positions will allow the network to possess minimum potential energy. Thus, at any given time instant, the positions of the cells can be determined by minimizing the potential energy of the system. Once a new daughter cell is produced, the original equilibrium balance is broken, and a new equilibrium can be calculated by minimizing the potential energy in the updated mass-spring network. Therefore, this process allows us to predict the migration of the cells through the embryonic development procedure.

### 4.3. Multi-Agent Cell Movement Simulation

**Figure 7.**
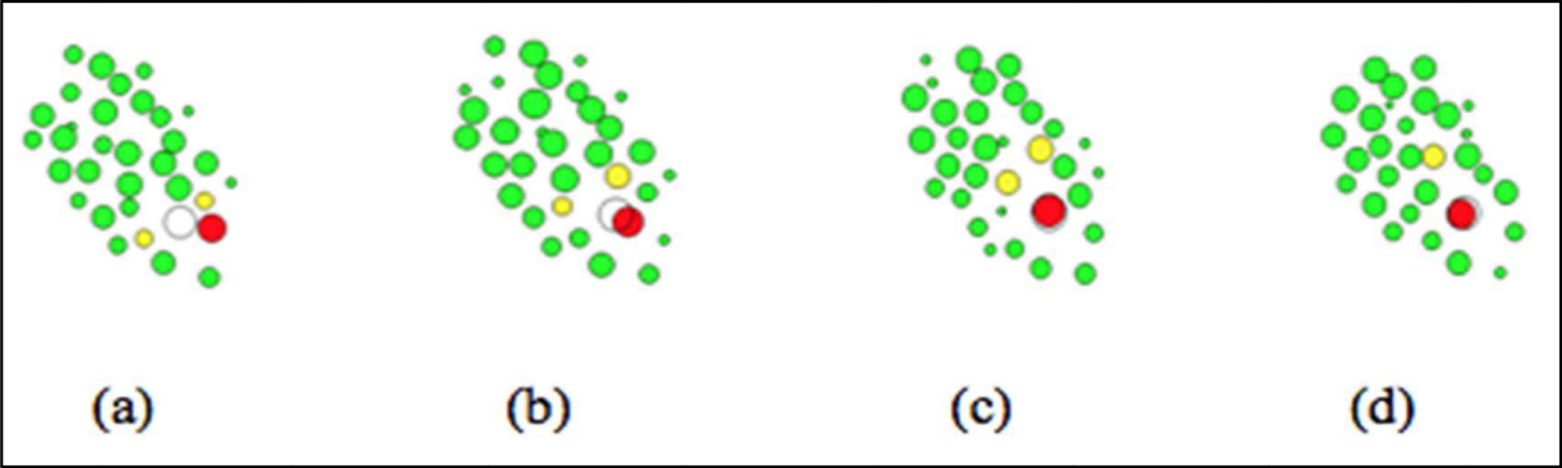
A hierarchical deep reinforcement learning model for the Cpaaa cell move-ment with four sub-goals, that is, to establish four special structure (Rosette) sequen-tially with its special neighbor cells (sub-goal cells) along the path. The white circle in each graph represents the observed destination of Cpaaa cell when the sub-goals are achieved sequentially. Red, yellow, and green cells represent the Cpaaa cell (in the training process), sub-goal cells at each migration phase, and other cells in images.

Our previous effort has shown the capability of deep reinforcement learning for modeling cell movement within an agent-based model [15]. Since the developmental phase in the early stage of C. elegans embryogenesis is regulated by a complex set of regulatory mechanism at various scales, the previous model that utilize the observational destination as a predefined dominant rule for the cell movement is a very strong regulation observed in the 3D live images. As an example, the Cpaaa cell migration path contains several phases, each is achieved via the establishment of a special biological pattern, called Rosette, with its neighbor cells along the path. With the above observations, we are working on a hierarchical deep reinforcement learning cell movement model in which the cell is controlled hierarchically by a set of sub-goals (Figure 7). Future plans for the cell movement modeling also include the design of multi-agent reinforcement learning [18] for the function group or even whole embryo, continuous control for output actions [19] of individual cells, division timing synchronization between the individual cells, as well as high performance simulation on parallel computing platform using asynchronous distributed model [20].

## 5. CONCLUSION

We presented a systematic approach to model the basic behaviors of individual cells, including cell fate, division, and movement, using 3D time-lapse images within an agent-based modeling framework. We summarized preliminary result and discussed the ongoing efforts and future directions for C. elegans embryo modeling, including an inferred developmental landscape for cell fate, a quasi-equilibrium model for cell division, and multi-agent, deep reinforcement learning for cell movement. The approach is a good fit for systematically investigation on individual cell’s dynamics and simulation-based hypothesis testing.
